# Supplementary material for: Glutamate decreases oxidative stress and lipid droplet formation in astrocytes
Source: J Cell Sci. 2025 Oct 9;138(19):jcs263983. doi: 10.1242/jcs.263983 (PMC12539204; doi:10.1242/jcs.263983)
Supplement: Supplementary information [file joces-138-263983-s1.pdf]

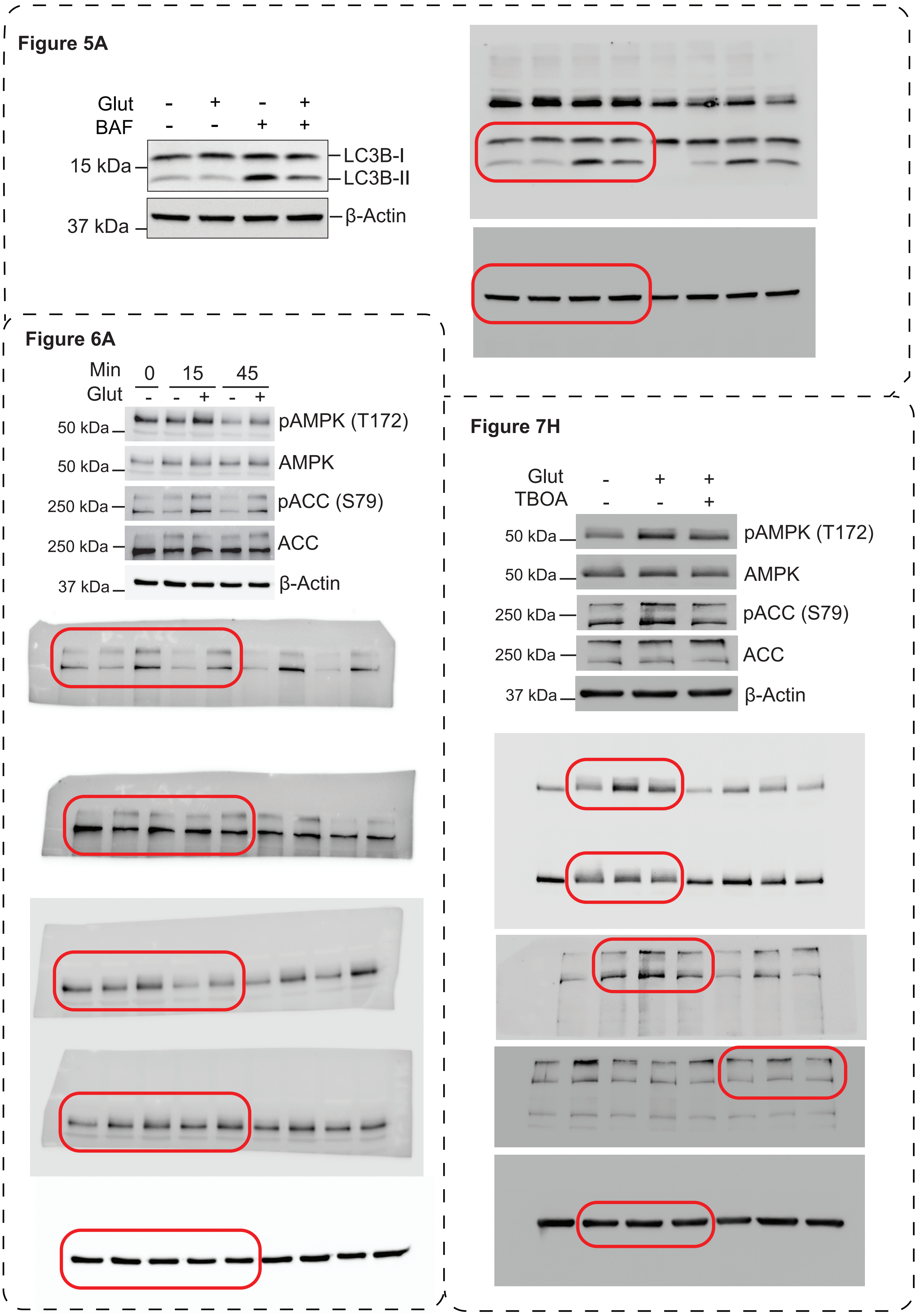

Rubio-Atonal et al. Figure S1

Fig. S1. Blot transparency.

Table S1. Lipidomics raw data

Available for download at
https://journals.biologists.com/jcs/article-lookup/doi/10.1242/jcs.263983#supplementary-data

Journal of Cell Science • Supplementary information
